# Embodied Emotion Regulation: The Influence of Implicit Emotional Compatibility on Creative Thinking

**DOI:** 10.3389/fpsyg.2020.01822

**Published:** 2020-08-06

**Authors:** Li Wu, Rong Huang, Zhe Wang, Jonathan Nimal Selvaraj, Liuqing Wei, Weiping Yang, Jianxin Chen

**Affiliations:** ^1^Department of Psychology, Faculty of Education, Hubei University, Wuhan, China; ^2^College of Life Science, Hubei University, Wuhan, China

**Keywords:** embodied emotions, emotional compatibility, emotional incompatibility, implicit emotions, creative thinking

## Abstract

The regulatory effect of embodied emotion on one’s general emotion and the impact of the compatibility or incompatibility of the two types of emotion on creative thinking are still debatable. The purpose of this study is to investigate these issues experimentally. In Experiment 1, participants completed an explicit positive and negative emotion test [Positive and Negative Affect Schedule (PANAS)] and an implicit positive and negative emotion test [Implicit Positive and Negative Affect Test (IPANAT)] twice on a computer after emotional video priming was used to induce negative emotions and facial expression manipulation was performed to induce embodied positive or negative emotions. It was found that maintaining the expression of a suppressed smile was helpful in regulating negative emotions (*p* = 0.047). Specifically, the implicit negative emotions induced by facial expression manipulation had a positive regulating effect on the implicit negative emotions induced by the video (T1, *M* = 47.813; to T2, *M* = 44.188). In Experiment 2, the positive or negative emotions of the participants were induced using emotional videos, and facial expression manipulation was used to induce their embodied positive or negative emotions. Then, the participants completed a creative test by completing alternative use tasks (AUTs) and Chinese character riddles. The AUT fluency score in the emotionally compatible group was significantly higher than that in the emotionally incompatible group (*p* = 0.032), but while experiencing negative emotions, the emotionally compatible group had a significantly higher originality score and insight in Chinese character riddle score than the emotionally incompatible group (*p* = 0.017, *p* = 0.004). Therefore, embodied negative emotion has a significant regulating effect on implicit negative emotion. The compatibility of emotion activated by facial expression and viewing a video contributes to creative thinking, whereas the incompatibility of emotion hinders creative thinking. The compatibility of emotion under positive emotions improved thinking fluency, whereas under negative emotions, it activated originality and insight in creative thinking. The influence of such emotional compatibility on creative thinking may be due to the regulating effect of embodied emotions on implicit emotions induced by emotional stimuli.

## Introduction

Creative thinking refers to the cognitive ability of an individual to create and develop new, valuable things. Creative thinking can also be defined as the cognitive ability to generate ideas, insights, and solutions that are original and flexible (Amabile, 1983; Craft et al., 2013). Fluency, originality, flexibility, and elaboration act as supportive roles in accelerating the creative thinking cognition process (Runco and Acar, 2012). The interactive role of emotions in the creative thinking process is one of the most intriguing research topics on creative thinking (Baas et al., 2008; Ding et al., 2015; Mastria et al., 2019).

Although the existence of a close relationship between emotions and creative thinking has been reported widely, there is no clear consensus on how these connections exist in the cognitive process. Studies have reported that individuals with positive emotions activated positive information connections and cognitive flexibility to enhance the fluency, originality, and insight; but individuals’ negative emotions had negative impact on the creative thinking process (Isen et al., 1986; Fernández-Abascal and Díaz, 2013; Mastria et al., 2019). However, some studies supported that negative emotions can stimulate an individual’s exploration of the real environment to improve the creative thinking process (Damian and Robins, 2012; Eastwood et al., 2012; Van Tilburg and Igou, 2012). Baas et al. (2013) proposed the dual pathway to creativity model, where approach-related traits such as positive affectivity and power motivation can accelerate creativity, as they enhance cognitive flexibility, but avoidance-related traits such as negative affectivity under the right circumstances do increase creativity, as they enhance cognitive persistence.

Embodied emotion theory indicates that emotional expression, perception, processing, and understanding are closely related to individuals’ physical arousal. Various aspects of cooperative relationships between physical actions and cognitive processes do exist in effective manner (Niedenthal, 2007; Glenberg et al., 2013). The muscle feedback signal transmitted by the body state triggers a unique neural activation pattern in the brain. These neural activation patterns represent unconscious emotions, and the embodied emotions belong to the category of implicit emotions (Strack and Deutsch, 2004; Wiers and Stacy, 2006; Neal and Chartrand, 2011). On the basis of the theory of embodied cognition, researchers have explored advanced cognitive activities, that is, the embodiment of creative thinking (Stanciu, 2015). On comparing the positive and negative emotional facial expressions, the positive and happy affect accelerated the flow of creative divergent thinking, whereas negative facial affect had no significant impact on creative thinking (Fernández-Abascal and Díaz, 2013).

An individuals’ emotions in daily life are quite complex. Emotions can be divided into explicit emotions, which the individual is aware of, and implicit emotions, which the individual is not aware of. Explicit and implicit emotions can form a compatible combination (i.e., the two emotional valences are consistent) and an incompatible combination (i.e., the two emotional valences are opposite). Studies have shown that different emotions being superimposed on each other will have a regulating effect on emotions overall (Gyurak et al., 2011; Koole and Rothermund, 2011). Riskind (1984) suggested that when an explicit negative emotion (failure experience) and an implicit negative emotion (slumped posture) formed a compatible emotion interaction, the feelings of helplessness and depression and motivation deficits are reduced, thereby playing a positive role in regulating negative emotions. Veenstra et al. (2017) reported that after inducing negative emotions, the stooped posture group showed mood recovery to a lesser extent than the upright and control groups, and the stooped posture created more negative thoughts than the other postures. The conflicting results of these two studies indicated that the intrinsic mechanism involved in the compatibility of embodied emotion and general emotion on emotion regulation needs further research.

Emotional compatibility or incompatibility has an important effect on creative thinking. A study showed that when participants’ emotional states were incompatible with the induced mood (low in depression with induced negative emotion), higher creative thinking was observed than in the compatible condition (low in depression with induced positive emotion) (Forgeard, 2011). Huang and Galinsky (2011) asked participants to complete the category association task under the incompatible conditions of recalling a happy thing when frowning and a sad thing when smiling and surprisingly found that participants showed greater innovation with new and unconventional answers for the problems. However, the theory proposed by Leung et al. (2014) believed that when an individual’s neuro-emotional traits and experienced emotions form a consistent experience, this consistency could promote performance of creative tasks. Another study also indicated that the compatibility between body posture and emotion was beneficial for creativity because the implicit emotions elicited by body posture matched explicit emotions, which in turn positively enhanced emotions on creativity, that is, through the flexibility or persistence pathway (Hao et al., 2017). The emotion matching hypothesis proposed that the emotions expressed by body postures had a special guiding effect on the self-regulation and information processing of the individual’s original emotions. This emotion matching effect was conducive to the regulation of emotions, which was consistent with the protective effect of emotions on individuals in the process of biological evolution (Riskind, 1982). This compatibility of emotion could generate a sense of coherence, which enhanced processing fluency and speeded up decision making of advanced cognition (Alter and Oppenheimer, 2006).

Based on earlier studies, research on the relationship between emotional compatibility and creative thinking is essential and needs further exploration. Many studies have ignored the combined effects of embodied emotions and emotions evoked by external emotional materials, and the impact of the interaction of these two emotions on high-level cognitive activities (such as creative thinking). In view of these concepts, this study explored the regulating effect of the interaction between embodied emotions (by facial expression manipulation technology) and emotions (by video activation). We also explored the impact of positive and negative emotional compatibility on the originality, fluency, and insight in creative thinking.

## Materials and Methods

### Experiment 1

In Experiment 1, we attempted to explore the regulating effect of the interaction between embodied emotions (activated by facial expression manipulation technology) and emotions (activated by emotional videos).

#### Participants

We calculated the sample size that was necessary to achieve 90% power (which is required to detect an adequate effect) *a priori* using G^∗^Power 3.1 (Faul et al., 2007). Based on *f* = 0.40 (Cohen, 1988), the power calculation yielded a recommended sample size of 45 participants. So 60 participants were selected initially, and they were undergraduate students from Hubei University. The participants were randomly divided into three groups (smile group, smile suppression group, and control group). Data from 12 participants who did not maintain their facial expressions as required were excluded. Thus, the final sample for the present experiment included 48 undergraduates with an average age of 20.46 years (*SD* = 2.19 years). The smile group, smile suppression group, and control group each had 16 participants. In the sample study, 13 students were male. All the students in the sample study were native Chinese speakers with normal hearing, normal vision or corrected vision, and no limb disabilities. Participants gave written informed consent prior to the experiment. The study was approved by the Institutional Ethics Committee at Hubei University.

#### Experimental Design

A 3 (expression control: smile group, smile suppression group, and control group) × 2 [emotion measurement: Time 1 (T1), Time 2 (T2)] mixed experimental design was used. The dependent variables were the scores and reaction times (RTs) of the Chinese version of the Implicit Positive and Negative Affect Test (IPANAT) and the Chinese version of the Positive and Negative Affect Schedule (PANAS), which were measured twice.

#### Experimental Materials

##### Emotion inductions

Participants watched short emotion-appropriate videos to induce corresponding emotion as per the procedures in previous studies (Forgas and East, 2008; Forgeard, 2011). The negative emotion-appropriate clips (3 min 13 s) were excerpted from movie clips “My Brothers and Sisters” (Jin et al., 2009). The film tells the story of an original happy family: because of the changes in the family, overnight, children become orphans and were separated.

##### Implicit positive and negative affect test

The IPANAT (Quirin et al., 2009) has a total of 36 entries and requires participants to evaluate the degree of association between six meaningless artificial compound words (SAFME, VIKES, TUNBA, TALEP, BELNI, and SUKOV) and six emotional words (six meaningless artificial compound words × 6 emotional words = 36 combinations) on a 4-point scale (1 = completely inconsistent; 2 = somewhat consistent; 3 = more consistent; and 4 = exact match); then, the scores of the six emotional words are calculated. The scores for the positive emotional words (cheerful, happy, and energetic) and negative emotional words (helpless, tense, and inhibited) indicate implicit positive affect (IPA) and implicit negative affect (INA), respectively. The higher the factor score is, the higher the implicit emotion score. A previous study showed that the Chinese version of the IPANAT holds better reliability and validity (Bao and Fu, 2018).

##### Positive and negative affect schedule

The PANAS (Watson et al., 1988) includes two factors, positive affect (PA) and negative affect (NA), each of which has nine entries. It requires subjects to indicate to what extent they are currently experiencing the emotions described by the presented words on a 5-point scale (1 = very slight or none at all; 5 = very strong). Studies have shown that the Chinese version of the PANAS has good reliability and validity (Bao and Fu, 2018).

#### Procedure

The experimental design was programmed with E-Prime 2.0 software to record the keys and RT data in the emotional test. Each participant completed the experiment in a separate compartment. There were 36 trials for implicit emotion using the Chinese version of the IPANAT. For each trial, the black “+” fixation point was presented for 250 ms, and then the matching task detection interface of a single artificial word and emotion word was presented. According to their first reaction, participants rated the degree of correspondence between the artificial words presented on the screen and the emotional words from level 1 to level 4 by pressing number keys 1, 2, 3, and 4. The Chinese version of the explicit emotion PANAS was administered similarly to the implicit emotion test, with a total of 18 trials and five rating levels that corresponded to number keys 1, 2, 3, 4, and 5 on the keyboard. To conceal the purpose of the current experiment, the participants were told before the experiment that it was an experiment on intuition.

**FIGURE 1:**
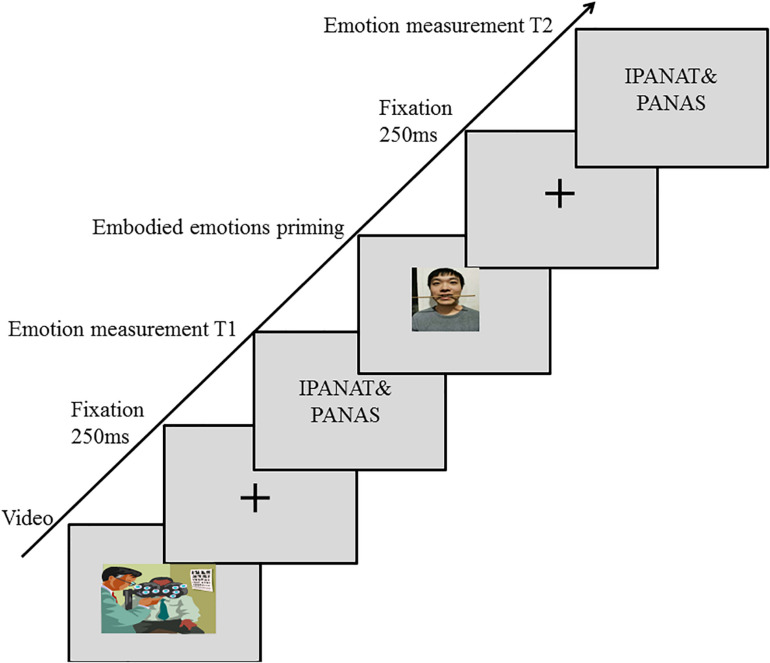
The trial structure of Experiment 1.

**FIGURE 2:**
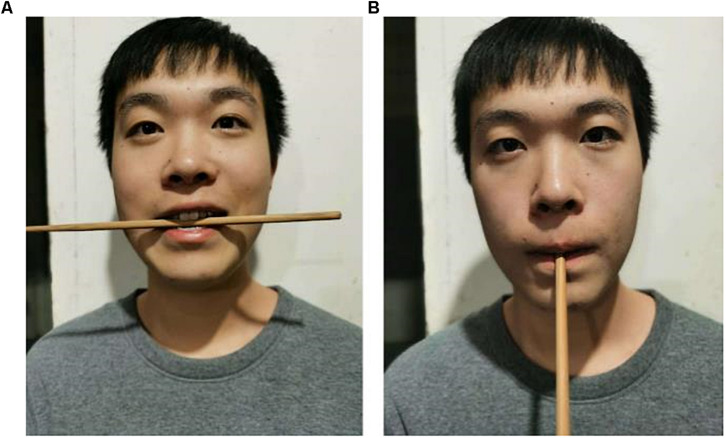
Facial expression manipulation. **(A)** smile group: biting chopsticks across the teeth. **(B)** Smile suppression group: with vertical chopsticks on the lips.

The experimental steps are shown in Figure 1. The participants had 2 min of practice under the guidance of the instructor before the formal test. The formal test required the participants to watch negative emotion videos for negative emotion activation, and then the first measurement (T1) of implicit emotions and explicit emotions was performed after the video watching was completed. Then according to the instructions presented on the computer screen, the instructor assisted the participants in completing the facial expression manipulation by using the facial muscle control paradigm to initiated embodied emotions (Wiswede et al., 2009; Figure 2). The positive emotion group held chopsticks horizontally in their teeth, which caused them to shape their faces into smiling, happy facial expressions. The negative emotion group held chopsticks vertically in their lips, which caused them to suppress their smiles and showed sad facial expressions. The control group did not control their facial expressions. Finally, the participants were asked to complete the measurement of implicit emotion and explicit emotion again while maintaining the standard expression control (T2).

#### Results and Discussion

##### Comparison of positive emotion score changes

**FIGURE 3:**
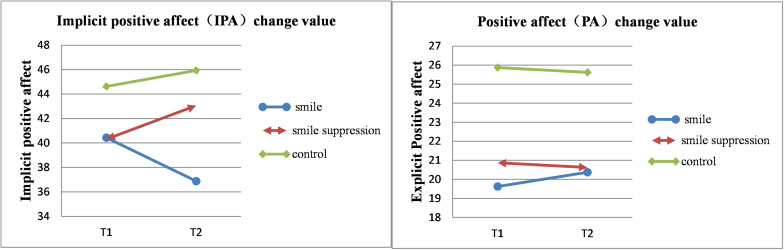
Left: Change value of IPA. Right: Change value of PA.

**FIGURE 4:**
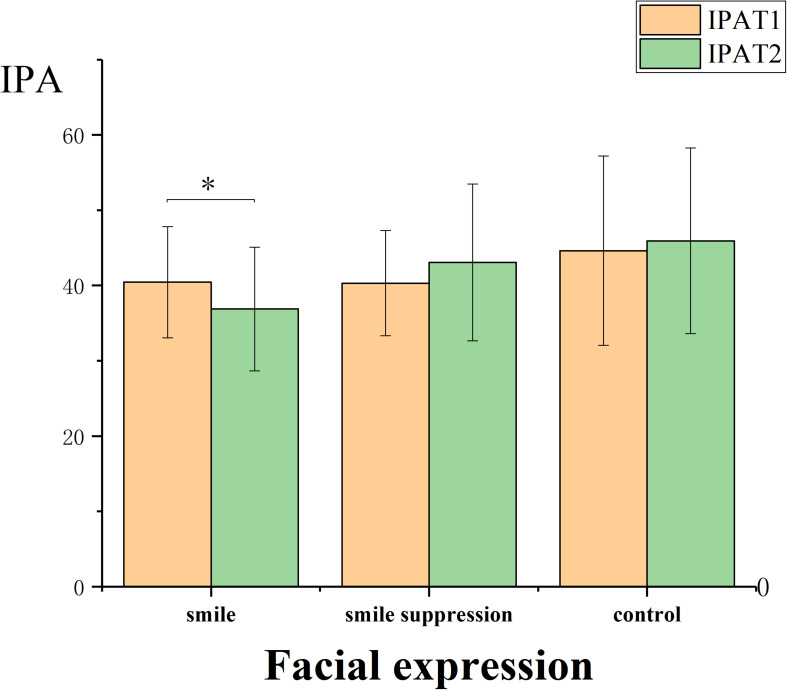
IPA of participants controlled by different facial expressions.

It was necessary to determine whether there was a regulating effect of embodied emotions induced by the manipulation of different facial expression on positive emotions. The descriptive statistical results are shown in Figure 3. The 2 ^∗^ 3 ANOVA with the time point (time: T1 vs. T2) of IPA and PA as within-subject factors and facial expression (expression control: smile vs. smile suppression group vs. control) as a between-subject factor was performed (Figure 4). The results showed that the main effects of measurement time [*F*(1, 45) = 0.028, *p* = 0.868] and facial expression [*F*(2, 45) = 2.038, *p* = 0.142] for IPA were not significant, but the interaction effect between measurement time and facial expression was significant [*F*(2, 45) = 3.675, *p* = 0.033, η*_*p*_*^2^ = 0.140]. A further simple-effects analysis found that there were significant changes in IPA between the pretest and posttest in the smile group. The IPA score decreased significantly [*F*(1, 45) = 4.260, *p* = 0.045] from T1 (*M* = 40.438, *SD* = 7.375) to T2 (*M* = 36.875, *SD* = 8.221). There was no significant difference in mood changes between the smile group [*F*(1, 45) = 2.540, *p* = 0.118] and the control group [*F*(1, 45) = 0.580, *p* = 0.451]. In the smile group, the participants’ IPA showed a decreasing trend, which means the embodied positive emotions activated by the smile facial expression manipulation did not play a positive role in regulating the negative emotions induced by the video but instead significantly increased the implicit negative emotions. However, the main effects of measurement time [*F*(1, 45) = 0.016, *p* = 0.899], facial expression [*F*(2, 45) = 3.063, *p* = 0.057], and the interaction effect [*F*(2, 45) = 0.261, *p* = 0.771] for PA were not significant. This suggests that expression manipulation had no regulating effect on the explicit emotion of the subjects.

##### Comparison of negative emotion score changes

**FIGURE 5:**
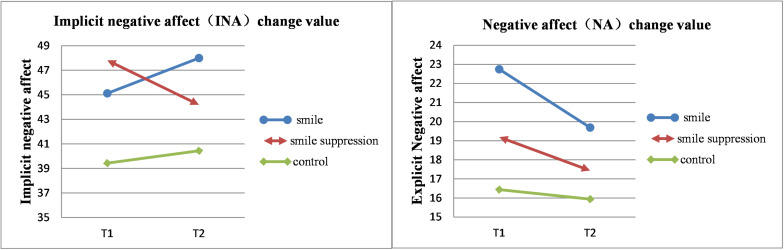
Left: Change value of INA. Right: Change value of NA.

**FIGURE 6:**
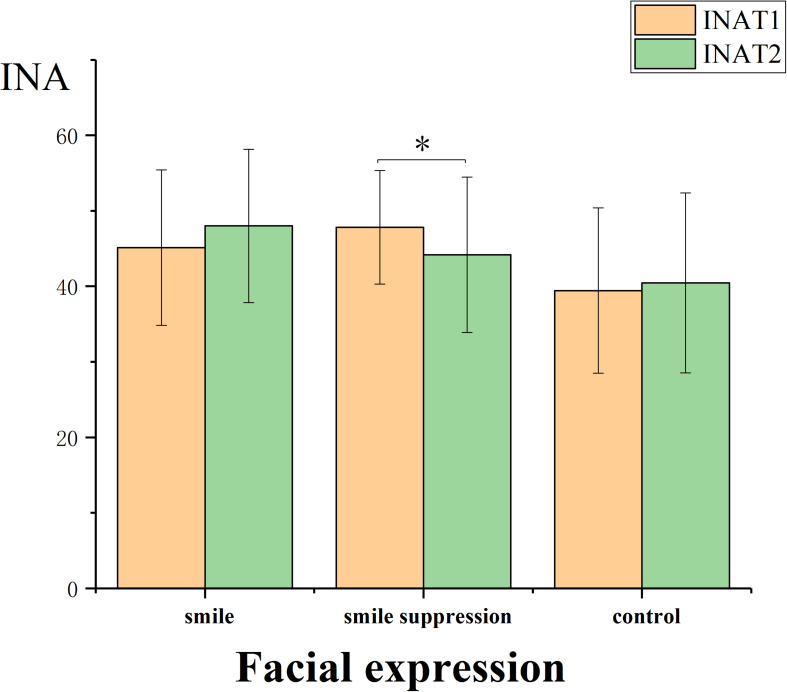
INA of participants controlled by different facial expressions.

It was necessary to determine whether there was a regulating effect of the manipulation of different facial expressions on negative emotions. The descriptive statistical results are shown in Figure 5. We performed a 2 ^∗^ 3 ANOVA of INA and NA with time point (time: T1 vs. T2) as a within-subject factor and facial expression (expression control: smile vs. smile suppression group vs. control) as a between-subject factor (Figure 6). The results showed that the main effects of measurement time [*F*(1, 45) = 0.007, *p* = 0.935] and facial expression [*F*(2, 45) = 2.320, *p* = 0.110] for INA were not significant. However, the interaction effect of measurement time and facial expression was significant [*F*(2, 45) = 3.570, *p* = 0.036, η*_*p*_*^2^ = 0.137]. A further simple-effects analysis found that there were significant changes in INA between the pretest and posttest in the smile suppression group. The INA score decreased significantly [*F*(1, 45) = 4.190, *p* = 0.047] from T1 (*M* = 47.813, *SD* = 7.530) to T2 (*M* = 44.188, *SD* = 10.297). There was no significant difference in the mood changes between the smile group [*F*(1, 45) = 2.640, *p* = 0.101] and the control group [*F*(1, 45) = 0.320, *p* = 0.575]. In the smile suppression group, the participants’ INA showed a decreasing trend, which means that the embodied negative emotions caused by the suppressing smile facial expression manipulation played a significant positive role in regulating the negative emotions induced by video, which in turn significantly alleviated implicit negative emotion. The main effects of measurement time [*F*(1, 45) = 4.480, *p* = 0.040, η*_*p*_*^2^ = 0.091] and facial expression [*F*(2, 45) = 3.507, *p* = 0.038, η*_*p*_*^2^ = 0.135] for NA were significant. This indicates that facial expression manipulation and PANAS scores are more sensitive to negative emotional states than to positive emotional states. The interaction effect [*F*(2, 45) = 0.782, *p* = 0.464] for NA was not significant.

##### Reaction time

The laboratory measurements of the Chinese version of IPANAT were similar to those of the implicit association tests (IATs) in the field of implicit cognition. In Experiment 1, artificial words were considered target concepts, and emotional words were treated as attribute concepts. When artificial words and emotional words appeared in pairs, the participants pressed keys to determine the degree of correspondence of emotional words and artificial words (e.g., SAFME-happy and SAFME-sad). The key RT was considered, and the key value was excluded. Data on implicit emotional reactions in the T1 and T2 measurements used the D-value calculations proposed by Greenwald et al. (2003) (Table 1).

**TABLE 1:** Compatible/incompatible reaction time (*ms*) and *D*-value.

		**Incompatible task group**		**Compatible task group**		**Effect**		***D*-value**		***D*-value**
		**(Artificial word – positive emotion)**		**(Artificial word – negative emotion)**						**Difference**
		**T1**	**T2**	**T1**	**T2**	**T1**	**T2**	**T1**	**T2**	
Smile group	*M*	2029.23	1725.21	1836.87	1623.53	192.36	101.68	0.17	0.1	0.07
(*n* = 16)	*SD*	1078.57	973.14	1218.37	996.4	1136.1	970.2			
Smile suppression group	*M*	1936.23	1725.73	1870.03	1709.1	66.2	16.63	0.13	0.02	0.11
(*n* = 16)	*SD*	524.81	637.25	524.33	824.46	517.13	724.9			
Control group	*M*	1784.01	1508.76	1722.23	1343.97	61.78	164.79	0.11	0.34	−0.23
(*n* = 16)	*SD*	713.79	553.14	446.54	417.75	586.52	489.39

1. The mean and standard deviation of the RT of each group at T1 were calculated. The RT for positive emotional words (e.g., SAFME-happy) in each group was longer than that for negative emotional words (e.g., SAFME-sad). The study showed that participants in each group preferred negative emotion words at T1 and T2 and that they were in a negative emotional state, which also indicated that emotion induction was effective. According to the difference between a compatible task and an incompatible task in IAT, *the artificial word–negative emotional word* pair was regarded as a compatible task, whereas *the artificial word–positive emotional word* pair was regarded as an incompatible task.
2. The D-value was calculated to reflect the participants’ preference level for negative emotion words. The D-value was the mean RT of positive emotion words (incompatible tasks) minus the mean RT of negative emotion words (compatible tasks), which was divided by the standard deviation of the total RT. The difference between the D-values of T1 and T2 was calculated. According to studies of implicit cognition, the difference in the D-value can be understood as the change in the preference level of negative emotion words. The comparison of the difference in D-values between the two groups showed that the D-value in the smile suppression group (D-value difference = 0.11) was higher than that in the smile group (D-value difference = 0.07) and control group (D-value difference = −0.23). From the perspective of RT, suppressing the expression of a smile could contribute to the positive regulation of negative emotions.

Based on the above analysis of the difference between the score changes of positive emotions and negative emotions and the changes in RT, it is reasonable to deduce that embodied negative emotions initiated by facial expression manipulation technology have a significant regulating effect on the implicit negative emotions initiated by video stimulation.

### Experiment 2

In Experiment 1, we found that allowing the participants to suppress the expression of a smile could effectively regulate their implicit negative emotion induced by the video. In other words, when the embodied emotional valence induced by body movement was consistent with the implicit emotion induced by the explicit emotion stimulus, the two compatible emotions had a positive regulating effect on the implicit emotions. To date, research on the relationship between emotion and creative thinking has not reached a consistent conclusion. In particular, the influence of embodied implicit emotion induced by an individual’s body movement on creative thinking has been ignored. The results of Experiment 1 showed that the compatibility of embodied emotions with the valence of emotions evoked by emotional videos could regulate implicit emotions. It is worth exploring whether the regulation of implicit emotions affects the creative thinking process, especially whether there are different influences on fluency, originality, and insight in creative thinking. Based on the results of Experiment 1, Experiment 2 was designed to further explore the impact of the compatibility of embodied emotions (facial emotion manipulation) with the emotions induced by external emotional stimuli (emotional video material) on creative thinking.

#### Participants

We calculated the sample size that was necessary to achieve 90% power (which is required to detect an adequate effect) *a priori* using G^∗^Power 3.1 (Faul et al., 2007). Based on *f* = 0.40 (Cohen, 1988), this power calculation yielded a recommended sample size of 68 participants. We thus required 80 undergraduate student volunteers from Hubei University to participate in this experiment. The participants were randomly divided into four groups (positive emotion-smile group, positive emotion-smile suppression group, negative emotion-smile group, and negative emotion-smile suppression group), and data from 12 participants who did not maintain their facial expressions as required were excluded. Therefore, the final sample for the study included 68 undergraduates with an average age of 20.01 years (*SD* = 1.92 years). In the overall sample, 27 of the students were males. There were 33 participants assigned to the emotional incompatibility condition and 35 assigned to the emotionally compatible condition; the assignments were random. Participants gave written informed consent prior to the experiment. The study protocol was approved by the Institutional Ethics Committee at Hubei University.

#### Experimental Design

A between-subject design of 2 (expression control: smile group, smile suppression group) × 2 (video emotion induction: positive, negative) was employed. The dependent variables were the scores for alternative use task (AUT) fluency, originality, and insight in Chinese character riddles.

#### Experimental Materials

##### Emotion induction videos

Participants watched short emotion-appropriate videos to induce corresponding negative or positive emotion as in previous studies (Forgas and East, 2008; Forgeard, 2011). The negative emotion-appropriate clips were the same as those in Experiment 1. The positive emotion-appropriate clips (3 min 28 s) were excerpted from movie clips of *Flirting Scholar* (Li et al., 2009). The film tells the interesting story of a Chinese scholar when he was a reading attendant at local officials in ancient times.

##### Alternative uses task

The AUT proposed by Guilford (1967) was adopted to measure participants’ creative thinking. Participants had to produce as many unusual or original uses as possible for common objects, such as paper clips (e.g., “making rings” and “cleaning nails”). The AUT is a reliable indicator of thinking fluency and originality in creative thinking (Runco and Mraz, 1992). Fluency scores were based on the total number of ideas given in the AUT. Originality scores were based on statistically infrequent responses. Scoring was conducted separately by two graduate students majoring in psychology. The answers generated by all participants were collected into a set. Synonyms were identified as the same answer. If the answer appeared to be novel (i.e., 5% or less of the participants in the sample gave the answer), a score of 1 was given, whereas any answer indicated by over 5% of the sample was marked as 0. The interrater agreement [intraclass correlation coefficients (ICCs) = 0.95] was satisfactory. Finally, the fluency and originality scores in solving two problems were averaged for each participant. In this study, the target task “chopsticks” was selected, and the participants were asked to write as many novel uses of chopsticks on the answer sheets as possible.

##### Insight in Chinese character riddles

Five pairs of Chinese character riddles were selected as materials from the Chinese Character Riddle Library (Zhen-Zhen et al., 2009), which were used to examine the “prototype inspiration effect” of insight. Qiu et al. believed that the use of Chinese character riddles as experimental materials could effectively explore the cognitive mechanism of insight in creative thinking through prototype inspiration under experimental conditions in Chinese individuals (Zhen-Zhen et al., 2008). Each pair of Chinese character riddles contained a prototype anagram (the prototype riddle was the prototype event in the prototype activation theory) and a target Chinese character riddle (the target riddle was the insight problem in the prototype activation theory). For example, if the prototype Chinese character riddle has a “十个兄弟,” then the correct answer is “克,” and if the target Chinese character riddle is “九个太阳,” then the answer is “旭.” The prototype heuristic rate (the correct rate with the prototype heuristic minus the correct rate without the prototype heuristic) was averaged to be 0.58. The scoring was separately conducted by two graduate students majoring in psychology. Each of the five target riddles was scored 1 point for each correct guess and 0 points for a wrong or no guess, and the total score for each correct answer was calculated.

#### Procedure

In the emotional initiation stage, the participants were required to focus on and watch *My Brothers and Sisters* (Jin et al., 2009) and *Flirting Scholar* (Li et al., 2009) to induce negative and positive emotions, respectively. Then according to the instructions presented on the computer screen, the instructor assisted the participants in completing the facial expression manipulation by using the facial muscle control paradigm to initiated embodied emotions (Wiswede et al., 2009; Figure 2). After the standard expression control was maintained, the AUT creativity test phase was performed. The participants were required to follow the main test guideline; that is, they should write as many new practical uses of “chopsticks” on the answer card within 3 min, and the answers should be realistic. During the entire process, the participants needed to maintain the expression with the chopsticks. They could take a 15-s break after the 3-min timer ended.

After the rest, the insight in Chinese character riddle task was conducted. The participants were required to learn five pairs of prototype riddles and target riddles first. There was no time limit. In this process, the instructor helped the participants understand the prototype riddle. After the participants fully understood the task, they were required to follow the instruction; that is, they should complete the other five pairs of Chinese character riddles quickly and accurately on the answer sheet within 3 min. Participants needed to maintain the expression manipulation with the chopstick during the entire process.

#### Results and Discussion

##### Fluency

**FIGURE 7:**
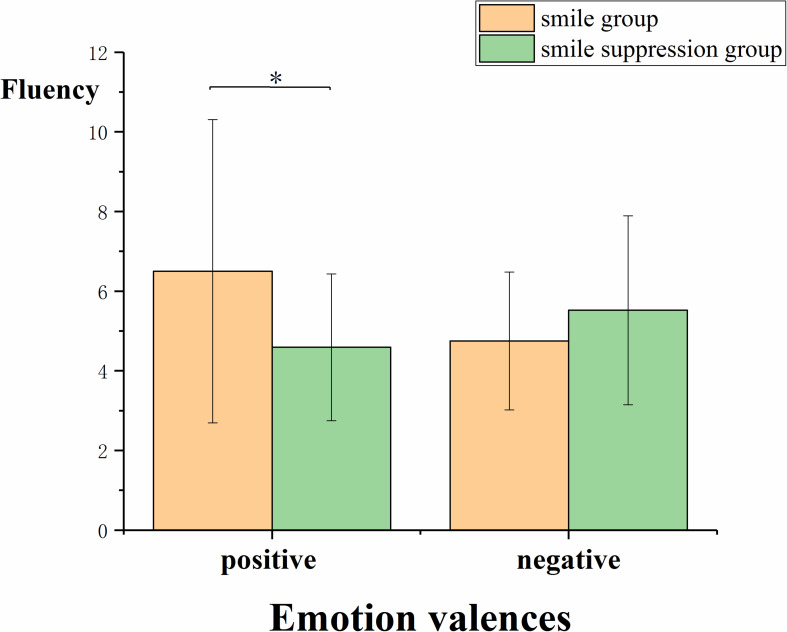
Fluency scores of participants under different emotion-induced conditions and expression manipulation states.

To analyze the effect of the compatibility of different facial expressions and different emotions on creative thinking, ANOVA was performed using facial expression manipulation and video emotion induction as independent variables and the thinking fluency score as the dependent variable (Figure 7). The results showed that the main effects of facial expressions [*F*(1, 64) = 0.799, *p* = 0.375] and explicit emotions [*F*(1, 64) = 0.408, *p* = 0.525] were not significant. However, the interaction between these two factors was significant [*F*(1, 64) = 4.512, *p* = 0.038, η*_*p*_*^2^ = 0.066]. Simple-effects analysis showed that for the condition in which positive emotion was induced by video, the fluency scores between the smile group and the smile suppression group were significantly different [*F*(1, 65) = 4.790, *p* = 0.032]. The fluency score in the facial smile group (*M* = 6.500) was significantly higher than that in the smile suppression group (*M* = 4.588). However, for the condition in which negative emotion was induced by the video, there was no significant difference in the fluency scores between the facial smile group and the smile suppression group [*F*(1, 65) = 0.720, *p* = 0.400]. This indicated that with positive emotions, the fluency of the group with a constant smile was better than that of the smile suppression group, which suggests that for positive emotions, emotional compatibility is conducive to generating the thinking fluency aspect of creative thinking.

##### Originality

**FIGURE 8:**
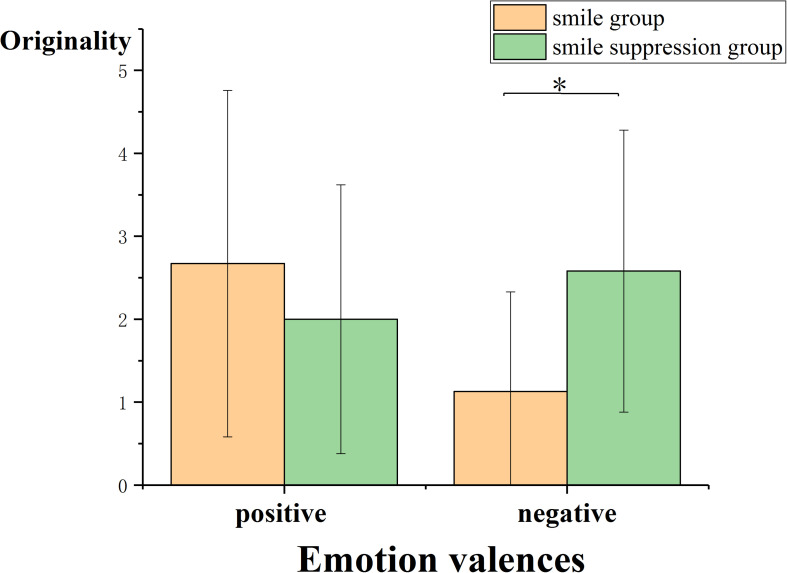
Originality scores of participants under different emotion-induced conditions and expression manipulation states.

To analyze the effect of the compatibility of different facial expressions and different emotions on creative thinking, ANOVA was performed using facial expression manipulation and video emotion induction as independent variables and thinking originality score as the dependent variable (Figure 8). The results found that the main effects of facial expressions [*F*(1, 64) = 0.937, *p* = 0.337] and explicit emotions [*F*(1, 64) = 1.342, *p* = 0.251] were not significant. However, the interaction between these two factors was significant [*F*(1, 64) = 6.699, *p* = 0.012, η*_*p*_*^2^ = 0.095]. Furthermore, simple-effects analysis showed that for the condition in which negative emotion was induced by video, the originality scores between the smile group and the smile suppression group were significantly different [*F*(1, 65) = 5.990, *p* = 0.017], and the originality score in the smile group (*M* = 1.125) was significantly lower than that in the smile suppression group (*M* = 2.588). However, for the condition in which positive emotion was induced by video, there was no significant difference in originality scores between the smile group and the smile suppression group [*F*(1, 65) = 1.40, *p* = 0.241. This indicated that with negative emotions, the originality of the smile suppression group was better than that of the smile group, which means that with negative emotions, emotional compatibility is conducive to generating the originality aspect of creative thinking.

##### Insight in Chinese character riddles

**FIGURE 9:**
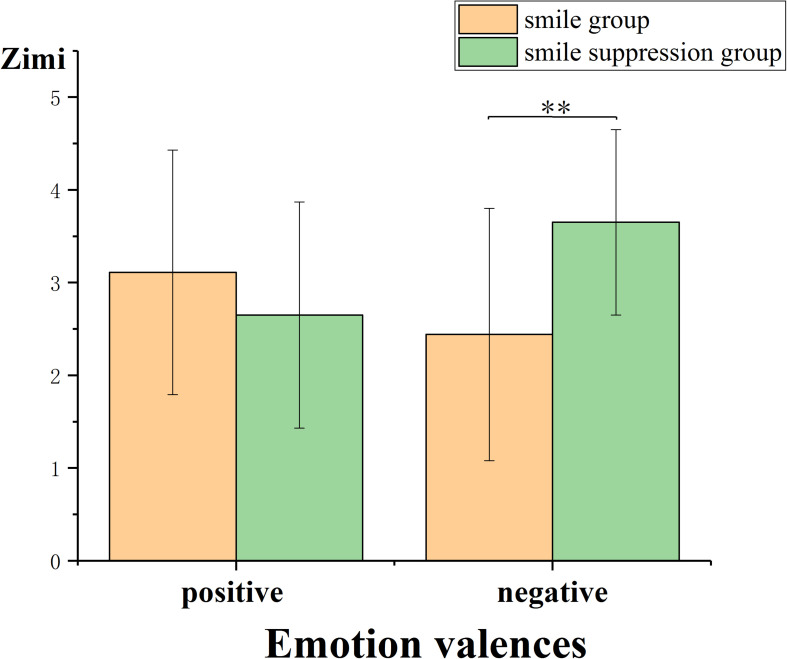
Insight Chinese characters riddle scores of participants under different emotion-induced conditions and expression manipulation states.

To analyze the effect of the compatibility of different facial expressions and different emotions on creative thinking, ANOVA was performed using facial expression manipulation and video emotion induction as independent variables and the insight Chinese character riddle score as the dependent variable (Figure 9). The results showed that the main effects of facial expressions [*F*(1, 64) = 1.687, *p* = 0.199] and explicit emotions [*F*(1, 64) = 0.323, *p* = 0.572] were not significant. However, the interaction between these two factors was significant [*F*(1, 64) = 8.500, *p* = 0.005, η*_*p*_*^2^ = 0.117]. Furthermore, simple-effects analysis showed that for the condition in which positive emotion was induced by the video, there were no significant differences in insight Chinese character riddle scores between the smile group and the smile suppression group [*F*(1, 65) = 1.330, *p* = 0.252]. However, for the condition in which negative emotion was induced by the video, the insight Chinese character riddle score in the smile group (*M* = 2.438) was significantly lower than that in the smile suppression group (*M* = 3.647) [*F*(1, 65) = 8.790, *p* = 0.004]. This indicated that with negative emotions, the insight of the smile suppression group was better than that of the smile group, which means that with negative emotions, emotional compatibility is conducive for generating the insight thinking aspect of creative thinking.

## General Discussion

In this study, we explored the impact of compatibility or incompatibility between emotions induced by facial expressions and emotional videos on creative thinking. In the first part of the study, the participants were measured based on implicit and explicit emotions after watching a negative emotion-inducing video, and then their implicit and explicit emotions were measured again after embodied positive emotions and negative emotions were induced through the manipulation of smiles or suppressed smile facial expressions. The results showed that for the smiling condition, implicit positive emotion decreased, and for the smile suppression condition, implicit negative emotion decreased, which proved that the compatibility of embodied emotion and video-induced implicit emotion has a positive regulating effect on implicit emotion. In the second part of the study, we proved that emotional compatibility promoted the flow of creative thinking, originality, and insight. Specifically, with positive emotions, the AUT fluency score of the emotionally compatible group (i.e., positive emotions/smiling expressions) was significantly higher than that of the emotionally incompatible group (i.e., positive emotions/smile suppression expressions). However, with negative emotions, the emotionally compatible group (i.e., negative emotions/smile suppression expressions) had a significantly higher originality score and insight in Chinese character riddle score than the emotionally incompatible group (i.e., negative emotion/smile expression). This suggests that the compatibility of emotion while experiencing positive emotions enhances the fluency of thinking, whereas the compatibility of emotion while experiencing negative emotions can accelerate the expression of originality and spiritual insight into creative thinking.

### Regulating Effect of Embodied Emotions on Emotions

In the first part of the study, embodied positive emotions did not play a positive role in regulating the negative emotions induced by video. Instead, the embodied negative emotions played a positive role in regulating the implicit negative emotions induced by the video, significantly relieving the negative implicit emotions. The experimental results suggested that the compatibility of emotions initiated by the video and embodied emotions initiated by facial expressions helped to regulate implicit emotions. Riskind (1984) and Veenstra et al. (2017) proved that body posture can affect emotional regulation. However, no consensus has been reached on which posture is more conducive to the recovery from negative emotions that have been induced. In Experiment 1, we explored the regulatory effect of embodied emotions induced by manipulating facial expressions on the negative emotions induced by videos, and our results were consistent with the “matching hypothesis” (Riskind, 1982). After negative emotions were experienced, the implicit negative emotion score of the participants who adopted the expression of suppressing a smile was lower than that of the smiling expression group. This indicates that the individuals immersed in negative emotion were not as sad as we imagined. When a person’s smiling expression “does not match” the current emotion (the person has just begun to feel sorrow), the smiling expression had an inhibitory effect on subsequent emotional recovery. The smile and suppressed smile expressions in this study played a special role in guiding and regulating the processing information of the individual. Smiling is a happy expression and more closely matched the participant’s emotional state (positive emotion). Suppressing a smile is similar to a sad expression and more closely matched the participant’s emotional state (negative emotion), which is more conducive to emotional regulation for emotionally compatible condition.

Fewer cognitive resources are consumed when emotions are better regulated, so creative thinking performance is better when experiencing emotional compatibility than when experiencing emotional incompatibility. The results of our study were inconsistent with those of Veenstra et al. (2017), as they used the self-reporting scale for emotional recovery measurements in their study. The emotions obtained through self-reporting may be distorted owing to social expectations, missing the implicit emotions that reflect the real emotions of the participants. The measurement of embodied implicit emotion should belong to the category of implicit emotions. We believe that indirect measurement was more suitable for measuring implicit emotions than self-reporting (direct measurement) because the autoactivated implicit emotion was only reflected in the explicit emotion scale when it was strong (Quirin et al., 2009). In view of this, in the first part of this study, we made participants evaluate emotionally neutral items and things that rarely have emotions attached to them, such as ancient Chinese hieroglyphs, Russian alphabet artificial words, or English alphabet artificial words. The degree of correlation was used to indirectly measure the regulation of implicit emotions induced by facial expressions on the emotions induced by video stimulation. Second, any external emotional stimulus not only can induce explicit conscious emotions but can also isolate implicit unconscious emotions behind conscious feelings (Clore and Ketelaar, 1997; Berridge, 1999; Kihlstrom et al., 2000; Winkielman and Berridge, 2004). In other words, in this study, the explicit emotion and the implicit emotion were further separated from the emotions induced by the emotional video. It was found that the embodied emotion induced by facial expression regulated the implicit emotion induced by the emotional stimulus, so it seemed that the measurement of emotion regulation tends to be highly accurate.

The performance of emotional compatibility to promote creative thinking can be explained by the characteristics of implicit emotion regulation. Implicit emotion regulation is effective and fast. Compared with explicit emotion regulation, implicit emotion regulation may be more effective. This is because the process of explicit emotion regulation requires effort and consumes certain self-control resources. When the individual’s self-control resources are insufficient, this regulation was not necessarily very effective. However, implicit emotion regulation does not require willpower; it is performed automatically and can be completed without conscious effort or supervision. This process may not consume any or only consume minimal self-control resources, so the process of implicit emotion regulation was more effective and occurs more easily (Koole and Rothermund, 2011; Etkin, 2014; Koole et al., 2015). When emotions are better regulated, fewer cognitive resources are consumed, and creative thinking performance when experiencing emotional compatibility is optimal.

### The Influence of Emotional Compatibility on Different Components of Creative Thinking

In the second part of our study (Experiment 2), we observed that emotional compatibility with positive emotions enhanced the fluency of creative thinking compared with that under emotional incompatibility. Emotional compatibility with negative emotions contributed to the originality and insight performance of creative thinking. The dual pathway to creativity model proposes that individuals can generate creative ideas, products, or solutions through either the cognitive flexibility path or the persistence path (De Dreu et al., 2008; Nijstad et al., 2010). Although both smiling expressions (positive emotions) and suppressed smile expressions (negative emotions) can enhance creative thinking, we believed that the cognitive mechanism that affects creative thinking under these two conditions might be different. According to the fluency dimension of creative thinking (see Figure 6), a smile under the positive emotional condition will further moderate the increment of negative emotions, and under positive emotional conditions, individuals can improve cognitive flexibility. According to Fredrickson’s extension-shaping theory of positive emotions (Fredrickson, 1998), from the perspective of evolution, positive emotions can provide a pleasant and safe situation to expand the attention range of individuals, thereby speeding up the response to new and different stimuli and ultimately the achievement of cognitive flexibility improvements. When experiencing positive emotions, more attention is paid to internal and subjective data, more cognitive resources are invested, and decisions involve less information about the external environment, resulting in more fluent and original ideas and opinions. Suppressed smile expressions in a negative emotional condition will further moderate the increment of negative emotions as well. Individuals with negative emotions are able to enhance the originality and insight aspects of creative thinking by improving their persistence with respect to thinking about problems (see Figures 7, 8). The mood-input model (Martin and Stoner, 1996) suggested that negative emotions can be regarded as signals of problems in the current environment (Schwarz, 2002), prompting individuals to remain fully alert (Forgas, 2007) and to make additional persistent efforts (George and Zhou, 2002). When experiencing negative emotions, individuals pay more attention to external and objective clues; in this study, the participants needed to suppress the interference of other information (such as riddle expression) when transitioning from the prototype riddle to the target riddle. However, once the direction of a possible breakthrough is determined, people will focus on a specific problem situation with more continuous efforts, ultimately achieving innovation.

### The Limitations of the Current Study and Directions for Future Studies

Owing to the complexity of creative thinking and emotions, the current study was only a preliminary exploration of the impact of embodied implicit emotions and explicit emotions on creative thinking. The evidence obtained through behavioral experiments was not sufficient. In future studies, we may use the electroencephalogram (EEG) or event-related potential (ERP) techniques to further explore the changes of neural activity in the brain area when the two emotions are compatible. Although the present study has found that the compatibility of embodied implicit emotions induced by facial expressions and explicit emotions induced by video stimulation has important effects on different components of creative thinking, there are still many factors that may influence creative thinking needed to investigate, such as individual personality, self-efficacy, and goal orientation.

Meanwhile, according to our results, the participants’ performance of originality and insight was better under the condition of negative emotion compatibility. In the future, we may need to extend this research to groups with prominent negative emotions – such as depressed individuals. Whether the depressed individuals can really regulate negative emotions through different body postures and further enhance creativity are worth discussing.

## Conclusion

The study showed that the embodied negative emotion activated by facial expression has a significant regulating effect on the implicit negative emotion activated by video stimulation. The compatibility of emotion activated by facial expression and video stimulation helps creative thinking, whereas the incompatibility of emotion hinders creative thinking. The compatibility of emotion with a positive emotional condition contributes to the fluency of thinking, whereas the compatibility of emotion with a negative emotional condition contributes to the originality and insight aspects of creative thinking. The influence of such emotional compatibility on creative thinking may be due to the regulating effect of embodied emotions on implicit emotions induced by emotional stimuli.

## Data Availability Statement

The datasets generated for this study can be found in the online repositories. The names of the repository/repositories and accession number(s) can be found in the article/Supplementary Material.

## Ethics Statement

Participants gave written informed consent prior to the experiment. The study was approved by the Institutional Ethics Committee at the Hubei University. Written informed consent was obtained from the individual for the publication of any potentially identifiable images or data included in this article.

## Author Contributions

LWu wrote the manuscript and analyzed the data. RH and ZW performed the experiments. JC and LWu conceived and designed the experiments. JS, WY, and LWe revised the manuscript and approved the final version of the manuscript. All authors contributed to the article and approved the submitted version.

## Conflict of Interest

The authors declare that the research was conducted in the absence of any commercial or financial relationships that could be construed as a potential conflict of interest.
